# Past hydroclimate extremes in Europe driven by Atlantic jet stream and recurrent weather patterns

**DOI:** 10.1038/s41561-025-01654-y

**Published:** 2025-02-27

**Authors:** Stefan Brönnimann, Jörg Franke, Veronika Valler, Ralf Hand, Eric Samakinwa, Elin Lundstad, Angela-Maria Burgdorf, Laura Lipfert, Lucas Pfister, Noemi Imfeld, Marco Rohrer

**Affiliations:** 1 Institute of Geography, University of Bern, Bern, Switzerland; 2 Oeschger Centre for Climate Change Research, University of Bern, Bern, Switzerland; 3 Climate Service Center Germany (GERICS), Helmholtz-Zentrum Hereon, Hamburg, Germany

**Keywords:** Atmospheric dynamics, Climate-change impacts

## Abstract

The jet stream over the Atlantic–European sector is relevant for weather and climate in Europe. It generates temperature extremes and steers moisture and flood-propelling weather systems to Europe or facilitates the development of atmospheric blocks, which can lead to drought. Ongoing climate change may alter the jet characteristics, affecting weather extremes. However, little is known about the past interannual-to-decadal variability of the jet stream. Here we analyse the strength, tilt and latitude of the Atlantic–European jet from 1421 to 2023 in an ensemble of monthly and daily reconstructions of atmospheric fields. We compare the variability of these jet indices with blocking frequency and cyclonic activity data and with drought and flood reconstructions in Europe. Summer drought is enhanced in Central Europe in periods with a poleward-shifted jet. An equatorward-shifted jet associated with decreased blocking leads to frequent floods in Western Europe and the Alps, particularly in winter. Recurrent weather patterns causing floods often characterize an entire season, such that an association between peak discharge and jet indices is seen on seasonal or even annual scales. Jet strength and tilt are significantly influenced by volcanic eruptions. Our 600-year perspective shows that recent changes in the jet indices are within the past variability and cannot be drivers of increasing flood and drought frequency.

## Abstract (web summary)

European climate extremes during the past 600 years were in large part driven by changes in the position and strength of the jet stream over the Atlantic, according to an analysis of terrestrial hydroclimate records.

## Main

**Extended Data Fig. 1:**
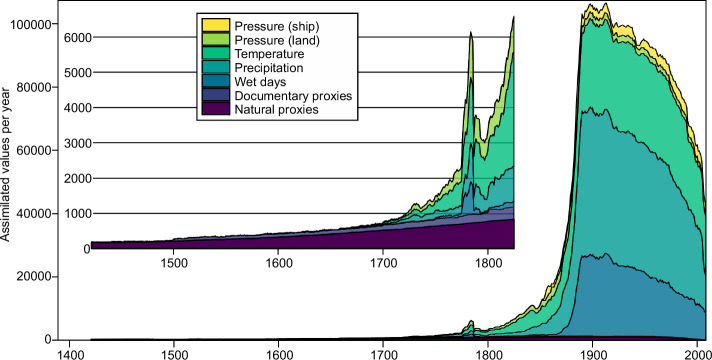
Observations assimilated into ModE-RA. Number of observations assimilated per year in the ModE-RA and ModE-RAclim data sets. For illustration, a series of tree ring width amounts to one observation per year, a monthly temperature record amounts to 12, a station record with monthly temperature, pressure, and precipitation to 36. After 1890, no new data series are added, and at the same time series that end are not replaced, therefore the number of assimilated observations does not increase further but decreases after 1890. Details on the generation of the data set and the evaluation are given in ref. ^15^, the observations are further described in refs. ^16,17^.

Because of its relevance for the surface climate^1–7^, several studies have aimed to reconstruct the past variability of the Atlantic–European jet stream^8–11^. Jet variations before around 1940 can only be diagnosed from its surface imprint due to insufficient upper-air data. Version 3 of the ‘Twentieth Century Reanalysis’ system (20CRv3)^12^, which assimilates surface and sea-level pressure (SLP) data, successfully reproduces the jet on synoptic to interannual timescales back to the early nineteenth century. Further back in time, palaeoclimate studies have made use of diverse sets of proxies ranging from Greenland ice cores to tree rings, with mixed success^8–11,13,14^. This is understandable given the sparseness of proxies, particularly over the Atlantic. Existing studies have used as few as two or three tree-ring chronologies^9,11^. Our study utilizes the palaeoclimate reanalysis Modern Era Reanalysis (ModE-RA)^15^. This dataset family provides monthly, global three-dimensional climate fields back to 1421 at 2° resolution. It is based on the offline assimilation of natural proxies, documentary data^16^ and instrumental observations^17^ into a set of atmospheric model simulations^18^. The number of observations assimilated is much higher than for other reconstructions, amounting to hundreds (seventeenth century), thousands (eighteenth century) or tens of thousands (nineteenth century) of values per year (Extended Data Fig. 1).

The observations are assimilated into 20 atmospheric model simulations (ModE-Sim)^18^ using prescribed monthly sea surface temperatures^19,20^ and exogenous forcings following the PMIP4 protocol^21^, that is, the fourth phase of the Paleoclimate Model Intercomparison Project. ModE-RA assimilates observations directly into the ModE-Sim ensemble and is the best estimate given both the forcings and the observations (Table 1). ModE-RAclim assimilates the same observations into a random sample of 100 realizations taken from all model years and members of ModE-Sim. It does not see a time-varying forcing, whereas ModE-Sim sees only the forcings but not the observations. For periods after 1806 and 1940 we also used, respectively, the 20CRv3 reanalysis^12^ and the European Reanalysis ERA5^22^ (that is, the latest climate reanalysis produced by the European Centre for Medium-Range Weather Forecasts). All analyses were performed per ensemble member except where noted.

**Table 1:** ModE-RA products

Product	*n*	Prior type	Obs
ModE-RA	20	Time-varying	Yes
ModE-RAclim	100	Invariant	Yes
ModE-Sim	20^a^	Time-varying	No

*n*, number of members. Obs, assimilation of observations? ^a^ModE-Sim has additional members not used for ModE-RA.

**Fig. 1:**
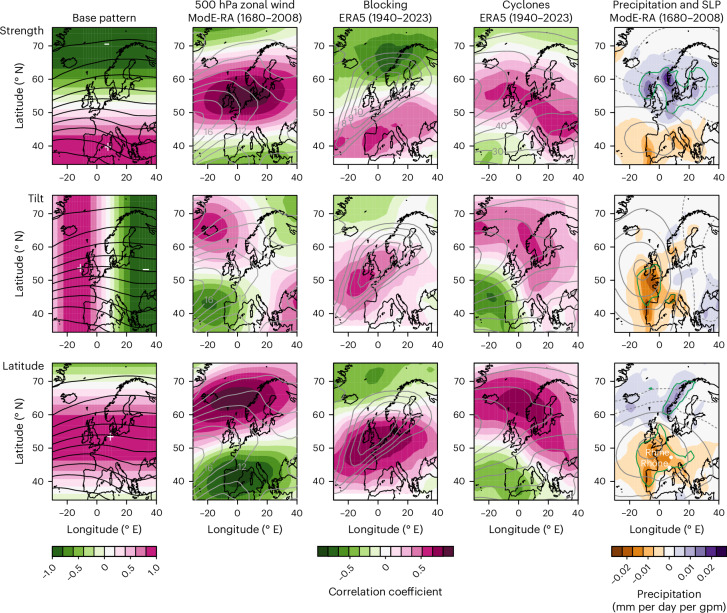
Climatic patterns associated with jet stream indices. Left column: base patterns used to define the strength, tilt and latitude of the jet. Solid lines indicate the climatological mean 500 hPa GPH in ModE-RA (50 gpm (geopotential metre) contour intervals). Second to fourth columns: correlation coefficients between the three indices and, respectively, 500 hPa zonal wind (contours indicate the climatological zonal wind in metres per second), the blocking frequency (%) or cyclonic activity (gpm) during the cold season. Significance (*P* = 0.05) corresponds to ±0.11 and ±0.21 for the ModE-RA and ERA5 analyses, respectively. Right column: regression coefficients of precipitation and the SLP (0.04 hPa per gpm contour interval centred around zero, negative values indicated by dashed lines) with cold-season-averaged jet indices (solid green contours denote that the correlation with precipitation is 0.4). The results are based on the ensemble mean.

**Extended Data Fig. 2:**
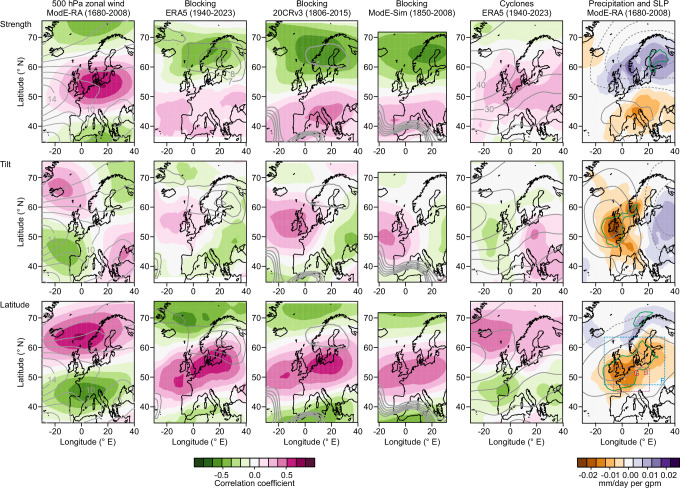
Patterns associated with jet stream indices in the warm season. (first to fifth column from left) Correlation coefficients between the three indices and 500 hPa u-wind (contours indicate climatological wind in m/s), blocking frequency (contours indicate climatological frequency starting at 7%, increment 1%) or cyclonic activity (gpm) in the warm season in different data sets. (right) Coefficient fields of regressions of precipitation and sea-level pressure (0.04 hPa/unit contour interval centred around zero, negative values dashed) onto the warm-season averaged jet indices, respectively (green: correlation with precipitation = 0.4). Dotted rectangles in the bottom right figure indicate the regions for the SPEI in Freund et al.^30^ (F), and scPDSI in Büntgen et al.^37^ (B). G marks the approximate position of the Glaser and Kahle^36^ SPI. Note that for illustration the results based on the ensemble mean are shown.

**Extended Data Fig. 3:**
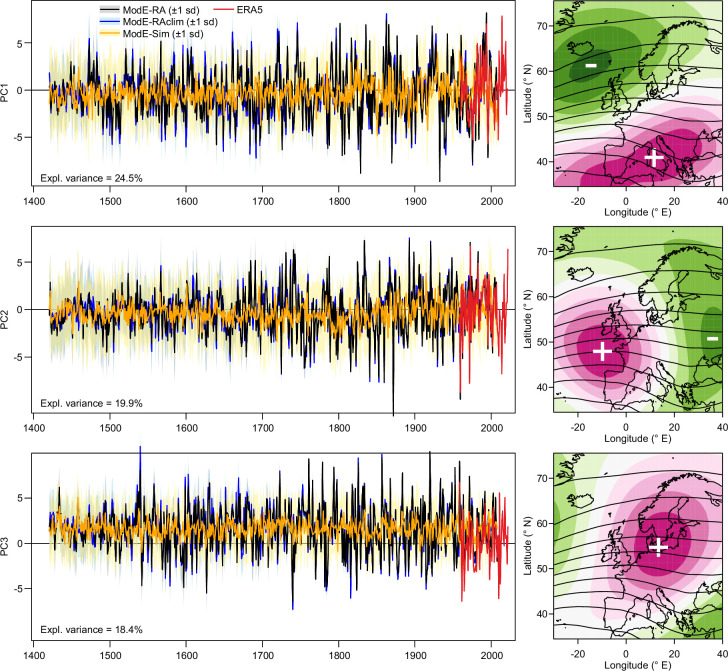
Principal Component Analysis approach for defining jet stream indices. (left) Time series and (right) patterns of the first three Principal Components of the deseasonalised monthly 500 hPa field over the Atlantic-European area calculated from one ensemble member over the 1850-1950 period (explained variances are indicated in the left plots). Time series were derived by projecting all members of all data sets onto these three patterns and then forming annual averages. Shading indicates ±1 standard deviation around the mean.

For the eddy-driven jet over the Atlantic–European sector (30° E to 40° W, 35–75° N), the indices of strength, tilt and latitude were defined as the coefficients of a latitude-weighted regression of deseasonalized fields of 500 hPa geopotential height (GPH) onto three predefined, orthogonal base patterns (Fig. 1 left column). Correlations of the indices with 500 hPa zonal wind from ModE-RA (Fig. 1 second column and Extended Data Fig. 2 for the cold and warm seasons, respectively) are up to ±0.9 and confirm that the base patterns indeed capture the strength (a monopole on the jet axis), tilt (a quadrupole around the jet axis describing its rotation) and latitude (a dipole north and south of the jet axis) of the jet stream. Principal component analysis gave similar results (Extended Data Fig. 3; Methods). For the following analyses we used the averages for November–April and May–October, which represent the cold and warm seasons, respectively.

The eddy-driven jet stream is a transient phenomenon that is expressed in synoptic features such as atmospheric blocks or cyclones, which affect hydroclimate variability over Europe. Therefore, we calculated atmospheric blocking (using an adapted^23^ Tibaldi–Molteni algorithm^24^) and cyclonic activity (using a 2.5–6 day bandpass filter^25^) in all members of 20CRv3, ERA5 and ModE-Sim^26^. Correlations of their seasonal means with jet indices are strong (up to ±0.8, Fig. 1 third and fourth columns), particularly in the cold season and for jet strength and latitude. A stronger jet is associated with more cyclonic activity in the storm track extension and an equatorward shift of blocking, whereas a poleward shift of the jet is associated with a poleward shift of cyclonic activity and an increase in blocking frequency. Similar patterns are found in 20CRv3 and ModE-Sim (Extended Data Fig. 2).

**Extended Data Fig. 4:**
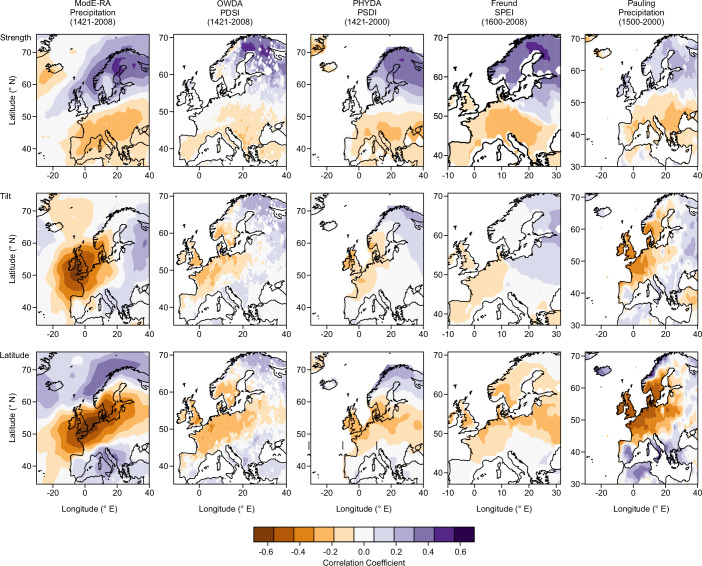
Imprint of ModE-RA jet indices in other reconstructions. Correlation fields of precipitation or drought variables from different reconstructions against jet strength, tilt, and latitude in ModE-RA (ensemble mean) for warm-season averages.

The mean precipitation in ModE-RA depends on the jet characteristics. During the cold season (Fig. 1, right column) it increases in the British Isles and Scandinavian region with a strengthened jet, and during both seasons it decreases in Western and Central Europe with a poleward-shifted jet (Extended Data Fig. 2). The patterns follow the inverse blocking frequency more than the cyclonic activity (this may be different for extremes) and are modified by the orography. Correlations of the warm-season ModE-RA jet latitude with precipitation or drought variables from other reconstructions^27–30^ (including an independent one^30^) confirm the results (Extended Data Fig. 4).

**Extended Data Fig. 5:**
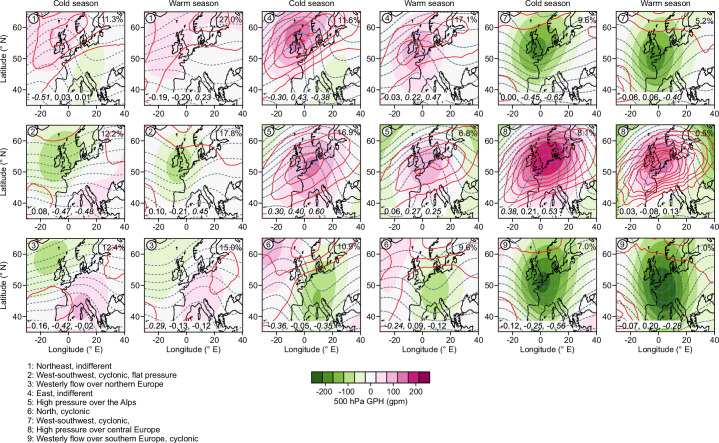
Daily weather types and blocking. Averaged 500 hPa geopotential height (dashed contours), anomalies (colours) and blocking frequency (red contours, increments 1% starting at 7%) for the nine CAP9 weather types (see lower left for description) in the cold and warm season, calculated in ERA5, 1940-2023. Percentage in the top right corner indicates the weather type frequency, the three numbers in the bottom left indicate the correlation of the seasonal weather types frequency with jet strength, tilt, and latitude, respectively (italics denote significant correlations at p = 0.05).

**Extended Data Fig. 6:**
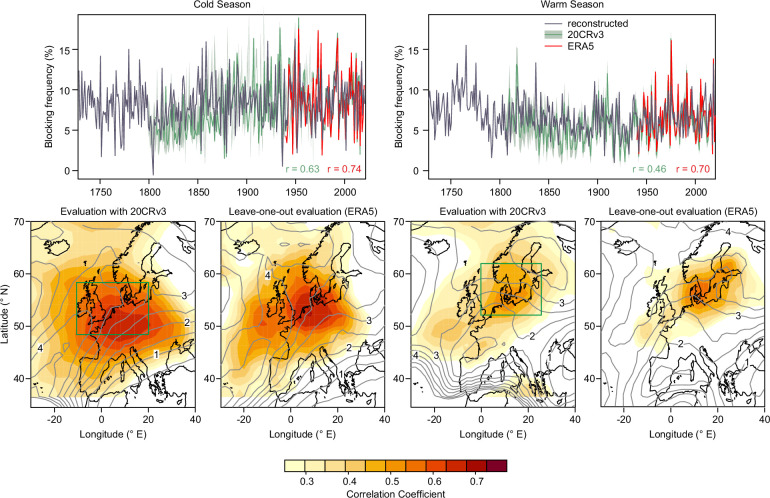
Reconstruction of blocking from weather type frequency. Reconstructed time series of blocking frequency as well as corresponding series from 20CRv3 (shading indicates ±1 ensemble standard deviation around the mean) and ERA5. The series relates to the average of the area indicated in the bottom figures (green rectangles). Bottom: Correlation coefficient and root mean squared error (%) of the spatial reconstruction of blocking frequency in independent evaluation, that is, in a leave-one-out evaluation as well as in a comparison with the 20CRv3 ensemble mean.

We address the daily scale back to 1728 using the CAP9 classification (cluster analysis of principal components with nine weather types) for Switzerland^31^. The types are associated with different blocking frequencies, and their frequencies are correlated with the jet indices (Extended Data Fig. 5). We used the seasonal weather-type frequency to reconstruct the seasonal blocking frequency using a grid-point-wise regression calibrated in ERA5 (Extended Data Fig. 6; Methods). Cyclonic activity leading to floods was addressed using a Flood Probability Index (FPI; see Methods) which weighs, for a given river, flood-prone weather types in a moving time window of five days. We calibrated the indices for seasonal peak discharge series for the Rhine in Basel^32^ and the Rhône in Beaucaire^33^ for the respective flood seasons (warm and cold).

## Six-hundred-year time series of jet indices

**Fig. 2:**
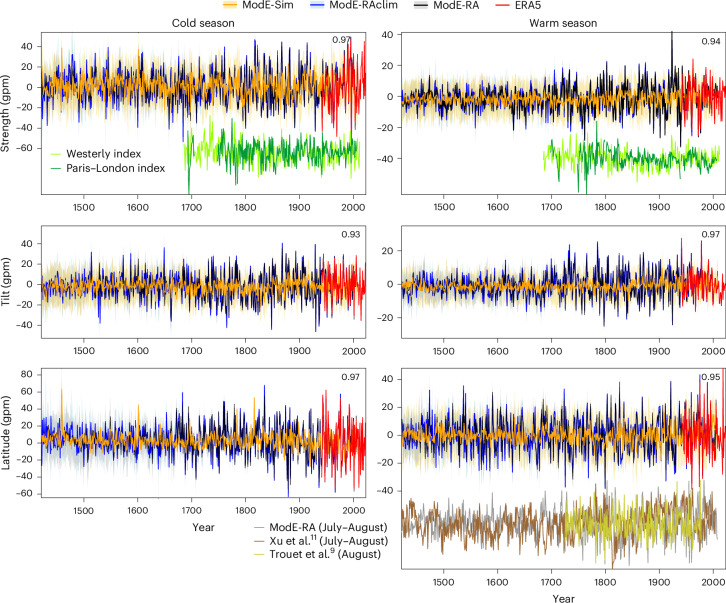
Reconstructions of jet stream indices. Time series of seasonal mean jet strength, tilt and latitude in different datasets. Shading indicates ±1 ensemble standard deviation. The numbers in the top right corner indicate the correlations between ModE-RA (ensemble mean) and ERA5. Existing reconstructions that correspond to the jet strength^10,34^ (light and dark green lines) and to the jet latitude in the warm season^9,11^ (grey, brown and yellow-green lines) are also shown. These series were standardized and are shown without scale.

The long time series in ModE-RA show an excellent agreement with ERA5 for both seasons (Fig. 2), indicating that the offline assimilation of surface data captures the jet characteristics well. Correlations between ModE-Sim and ModE-RA are weak (average 0.18), although significant, indicating that only a little of the jet variability is explainable by sea surface temperatures or external forcings, in agreement with other studies^7^. Conversely, average correlations between ModE-RAclim and ModE-RA are 0.91 as the North Atlantic–European region is well constrained by observations^15^. The variability in the ensemble mean decreases back in time when reconstructions are less well constrained. The ensemble spread in ModE-RAclim is relatively low back to 1421 for the warm season, whereas in the cold season there is a change in the ensemble spread around 1680, when several instrumental series start. This is because only few documentary data series cover the cold season and no tree-ring proxies are assimilated in the cold season. Therefore, we restrict some of our analyses to the post-1680 period.

How do the time series compare with other reconstructions? The jet strength index can be compared with the westerly index^10^ (the percentage of days with westerly winds from ship-based observations) in the English Channel back to 1684 or with the SLP difference between Paris and London^34^ back to 1692 (Fig. 2), although these are not independent of ModE-RA. Correlations with the ensemble mean jet strength are 0.58 (westerly index) and 0.38 (Paris–London index) for the cold season. They are higher, 0.64 and 0.67, respectively, when restricting the season to December–March, and even higher, at 0.68 and 0.74, when using our first principal component (PC) series over December–March. Even though these indices do not measure the same phenomena, they are closely associated and indicate reproducibility. During the warm season, correlations with the jet strength (at 0.23 (westerly index) and 0.17 (Paris–London index)) and the first PC series (0.32 and 0.30, respectively) are lower, although they remain highly significant.

**Extended Data Fig. 7:**
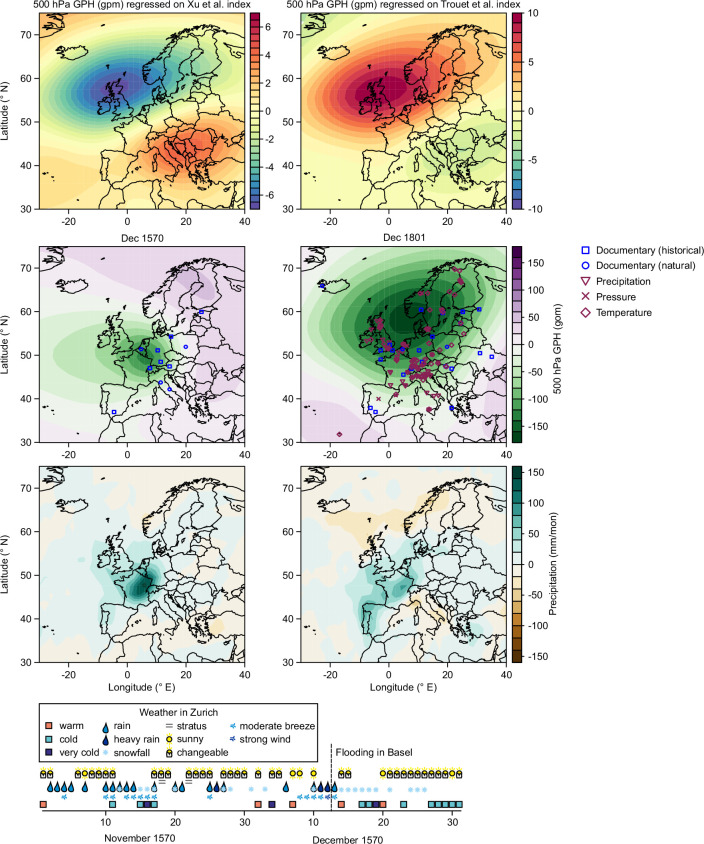
Patterns of 500 hPa geopotential height for two tree-ring based jet latitude reconstructions and for two winter flood cases. (top) Regression coefficient of 500 hPa GPH from ModE-RA since 1422 (in gpm) in Jul.-Aug. (left) and Aug. (right) onto the jet latitude reconstructions by Xu et al.^11^ (left) and Trouet et al.^9^ (right), respectively. (second and third row) 500 hPa geopotential height and precipitation anomaly (relative to 1422-1850) in Dec. 1570 and Dec. 1801. Symbols show the observations that went into the reconstruction. (bottom) Daily weather in Zurich in Nov. and Dec. 1570 observed by Wolfgang Haller.

The jet latitude during the summer (defined on the basis of 300 hPa wind) has been reconstructed from two tree-ring chronologies for August by Trouet et al.^9^ and from three tree-ring chronologies for July–August by Xu et al.^11^ (Fig. 2). The former reconstruction (termed the latitude of the North Atlantic jet by the authors^9^) correlates well with our jet latitude index (0.42). The latter reconstruction (called the jet stream latitude in the North Atlantic–European sector^11^) is, however, negatively correlated with the former (−0.67) and negatively correlated with our jet latitude index (−0.16). It drops strongly following the volcanic eruptions of the early nineteenth century, similar to the temperature, which our series do not. These discrepancies arise from different definitions. Regression maps of the ModE-RA 500 hPa GPH onto these indices (Extended Data Fig. 7) show almost opposite patterns. The index of Xu et al.^11^ is associated with a poleward shift of circulation (including the subtropical jet) over southeastern Europe but an equatorward shift of the eddy-driven jet over the Atlantic, whereas the index of Trouet et al.^9^ is associated with a poleward shift of the eddy-driven jet over the eastern North Atlantic. This indicates that care should be taken when using the term ‘jet latitude’. Our jet latitude index describes the northward or southward shifts of the axis of the strongest mean zonal wind at 500 hPa (Fig. 1), reflecting the eddy-driven jet.

## Jet characteristics and hydroclimate extremes

During the summer, we expect low precipitation and hence drought proneness in Central Europe during situations with a poleward-shifted jet. Conversely, flood proneness is expected in situations with an equatorward-shifted jet, which will affect both winter floods (for example, in the Rhône catchment) and summer floods (in Alpine catchments)^35^. For these regions, we analysed whether there is a relation between the jet characteristics and hydroclimatic extremes using additional datasets on floods and droughts. Flood proneness in southern Scandinavia during the cold season (due to a strengthened jet) is not addressed because of the strong effect of snow melt on floods.

**Fig. 3:**
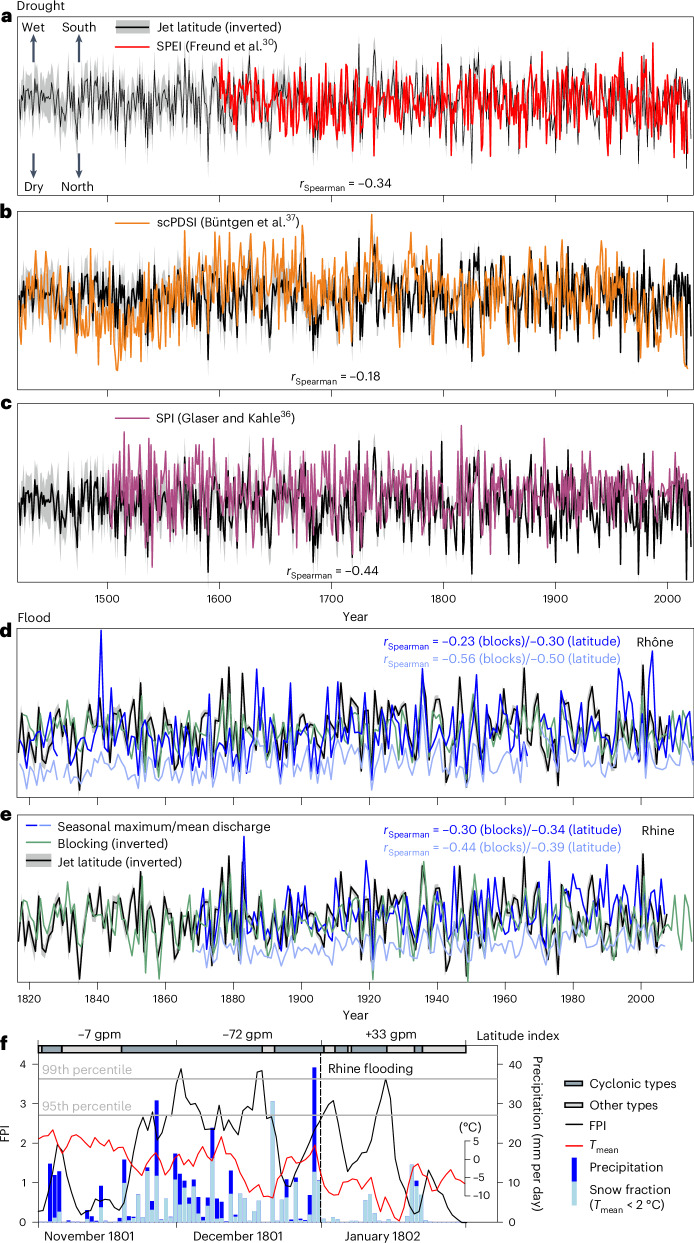
Jet stream latitude and indicators of drought and flooding. **a**–**c**, Time series of the May–August jet latitude (where the solid line and shaded area denote the ensemble mean ±1 standard deviation) compared with the SPEI reconstructions averaged over Europe (**a**), the scPDSI reconstructions averaged over Central Europe (**b**) and the SPI reconstructions for Germany (**c**). **d**,**e**, Time series of the November–April jet latitude and Central European blocking frequency compared with the mean and maximum discharge data of the Rhine in Basel (**d**) and of the Rhône at Beaucaire (**e**); the *y* axes are arbitrary. For the averaging regions see Extended Data Fig. 2. **f**, FPI (left axis; black solid line) for Basel from November 1801 to January 1802. Also shown for this period are the daily precipitation data (right axis; blue bars, where precipitation on days with a daily mean temperature (*T*_mean_) below 2 °C, evaluated on a 1 × 1 km scale^41^, is marked as the potential snow fraction) and the temperature (inset axis; red solid line) averaged over the Swiss catchment of the Rhine. Dark and light grey horizontal bars indicate cyclonic (types 2, 6, 7 and 9; Extended Data Fig. 5) and other CAP9 weather types, respectively. Numbers shown indicate the monthly jet latitude index.

Most drought reconstructions are not completely independent from ModE-RA as they share common tree-ring or documentary input data^27–29,36^. The only completely independent reconstructions are field reconstructions using the Standardized Precipitation–Evapotranspiration Index (SPEI)^30^ (which we averaged over the area most strongly affected by jet latitude in ModE-RA) and an area-averaged self-calibrating Palmer Drought Severity Index (scPDSI) reconstruction for Central Europe (Fig. 3)^37^, both of which are based on stable isotopes in tree rings. We compared these series with the May–August jet latitude as this corresponds best with the seasons captured by the drought indices. The Spearman correlation (*r*_Spearman_) values are −0.34 for the SPEI reconstruction (Fig. 3a) and −0.18 for the scPDSI reconstruction (Fig. 3b). Both are significant (*P* < 0.05). Note, however, that both reconstructions may be imperfect. We also compared our jet indices with a recent reconstruction of the Standardized Precipitation Index (SPI) in Germany^36^, which is, however, not fully independent (Fig. 3d). We used the SPI4 (SPI for four months) for August, which is affected by the May–August precipitation, and correlated this with the May–August jet latitude. The Spearman correlation (−0.44) indicates a good correspondence.

**Extended Data Fig. 8:**
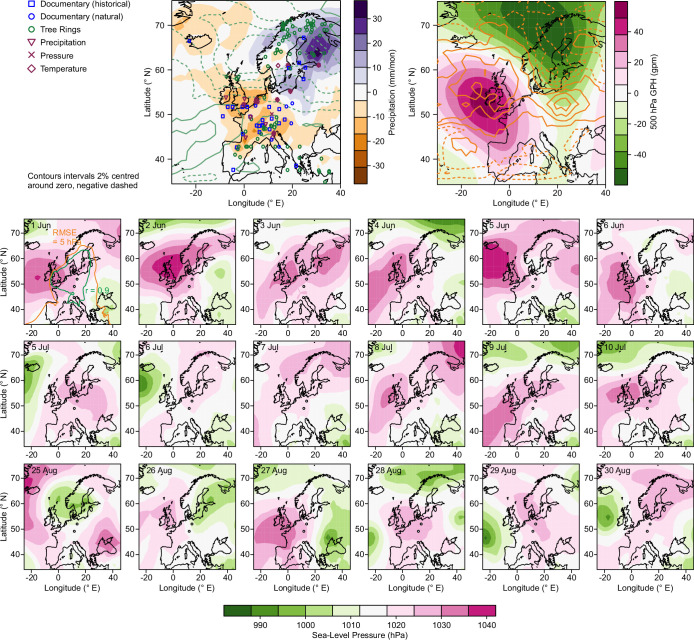
Analyses of the dry summer of 1741. (top) Anomalies (with respect to 1728-1757) of total precipitation, 500 hPa GPH, and blocking frequency (contours, left: regression approach based on CAP9, right: analogue method based on ERA5) in Jun.-Aug. 1741. Symbols show the observations on which the reconstructions are based. Bottom: Sea-level pressure from an analogue reconstruction for three periods of 6 days that were dominated by weather types 3, 4, 5, and 8, which are often associated with ridges or blocking. The contours in the figure for 1 June indicate the daily skill (RMSE, correlation) of the analogue approach in an independent evaluation within ERA5 in which the year 1940 was predicted from 1941-2023.

We analyse the three summers (June–August) of 1741, 1800 and 1802—for which strong drought conditions were reported^36^—in more detail using daily data. These are not well studied as they occurred before the start of 20CRv3. In the summer of 1741 (Extended Data Fig. 8), the jet latitude was increased in ModE-RA, associated with a positive tilt. Daily CAP9 data show an 81% increase in weather types 3, 4, 5 and 8 (those associated with ridging and blocking) compared with the period 1728–1757. These types occurred in five episodes. For three periods of six days each, daily SLP reconstructions from an analogue approach are shown (Extended Data Fig. 8), indicating high-pressure ridges forming anticyclones. The blocking frequency reconstructed via CAP9 was increased over southwestern Europe and the Alps. The blocking frequency in the analogue days was increased over Western and Central Europe. These analyses show that the seasonal mean position of the jet was related to recurrent anticyclonic weather patterns and blocks^38^. The other extreme drought summers studied, that is, 1800 and 1802 (not shown), were related to persistent anticyclonic conditions, with few very long episodes of ridging and blocking in CAP9 of 28 and 31 days, respectively. In addition to atmospheric circulation, many other factors, such as atmospheric moisture content, soil moisture and land–surface feedbacks, contribute to drought^39^.

For river floods, the situation is more difficult than for droughts as the former predominantly manifest as short events. Furthermore, factors other than the atmospheric circulation—snow melt, atmospheric moisture content, static stability or various hydrological factors—play an important role. Before analysing floods directly, we compared the jet indices with the seasonal 75th percentile of the FPI^35^. The strong correlations of −0.66 and −0.45 with the jet latitude for the Rhône and the Rhine, respectively, confirm that the jet latitude is an important driver of flood proneness.

From the longest daily discharge record, for the Rhône in Beaucaire back to 1817, we calculated the seasonal mean and seasonal maximum discharge. Cold-season correlations with the jet latitude and with blocking over Central Europe are strong (Fig. 3d), particularly for the seasonal mean discharge (*r*_Spearman_ = −0.56). We also find significant negative correlations between the seasonal maximum discharge and the seasonal mean blocking frequency and jet latitude. Strong cold-season relations are found also for the Rhine in Basel (Fig. 3e), where daily discharge data go back to 1869.

The time series of annual peak discharge for the Rhine in Basel reaches back to 1808^32^ and documentary sources provide a list of floods along with dates back to the thirteenth century. We analysed monthly jet indices and 500 hPa GPH fields for all flood events after 1421 that were characterized as either severe or catastrophic^32,40^. The jet latitude index in these months was negative on average, particularly for the winter flood events, where it averaged to −33 gpm or −1.1 standard deviations (11 out of 12, including all four ‘catastrophic’ events^40^, had a negative index). The catastrophic December floods of 1570 and 1801 were analysed in more detail.

For the flooding on 31 December 1801 (Fig. 3f), the daily FPI series was above the 95th percentile during a large part of the preceding month, even surpassing the 99th percentile on three days. An exceptionally long sequence of cyclonic weather types lasted from mid-November into January. The jet latitude index was extremely negative in December 1801, and the 500 hPa GPH anomaly field (Extended Data Fig. 7) shows strong troughing. We averaged the gridded (1 × 1 km) daily precipitation and temperature reconstructions^41^ for the Swiss Rhine catchment and found several strong precipitation events in December 1801. A significant fraction of the precipitation may have fallen as snow. The temperature then increased at the end of December, arguably melting some of the snow. Extreme precipitation on 30 December 1801, occurring with a high zero-degree line, eventually led to the flood event.

For the December 1570 event the monthly 500 hPa GPH anomaly indicates a southward-shifted jet. Daily weather data for Zurich (the records of Wolfgang Haller spanning 1545–1576)^42^ suggest a succession of cyclones in the 30–40 days preceding the event, bringing windy and rainy or snowy weather (Extended Data Fig. 7). After a possibly warmer phase, several days of heavy precipitation triggered the flood on 12 December 1570. In Haller’s notes, two days had heavy rainfall. A chronicler in Geneva even writes of five days of intense rainfall (“day and night”)^32^.

The two winter cases show that flood-prone weather sequences may characterize an entire season. Therefore, the association between jet characteristics and peak discharge translates to longer timescales, as seen in the seasonal correlations in Fig. 3d,e. Comparing the annual peak discharge with the annual mean jet latitude, we find significant correlations of −0.24 for the Rhône in Beaucaire back to 1817 and −0.19 for the Rhine in Basel back to 1808.

## Drivers of jet characteristics

**Extended Data Fig. 9:**
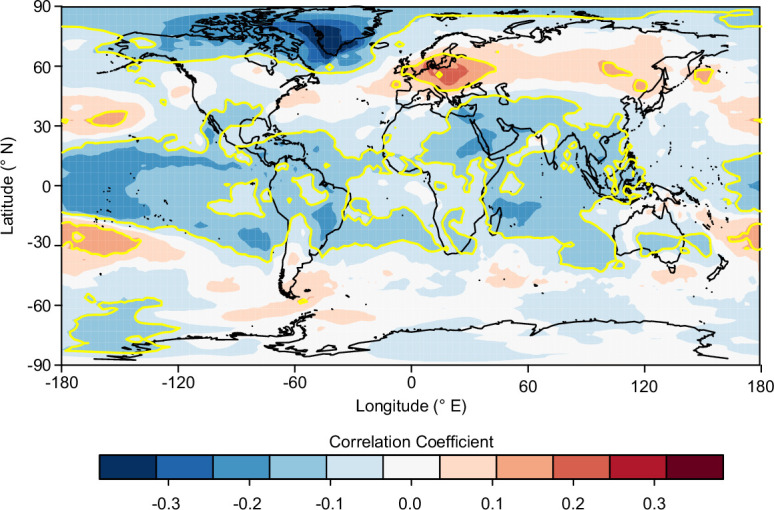
Effect of El Niño/Southern Oscillation on Atlantic-European jet latitude. Pearson correlation between Nov.-Apr. jet latitude and the Jul.-Dec. global temperature field (that is, temperature leads the jet by 4 months, yellow line indicates p = 0.05). Note that the correlation is shown for ensemble mean (individual members give very similar results), and for the period after 1680.

We analysed the possible drivers of the jet indices. Specifically, we correlated the cold-season jet latitude with the global near-surface temperature in ModE-RA four months earlier (July–December, that is, the onset and maturing phase of an El Niño–Southern Oscillation event) to test whether there is predictability. We restricted the analysis to 1680–2008 and used the ensemble mean. The resulting map (Extended Data Fig. 9) shows a negative correlation for the central tropical Pacific, which, although weak, is statistically significant. This is consistent with previous findings on El Niño–Southern Oscillation influences on the Atlantic–European sector that find weak but significant effects^43,44^.

**Fig. 4:**
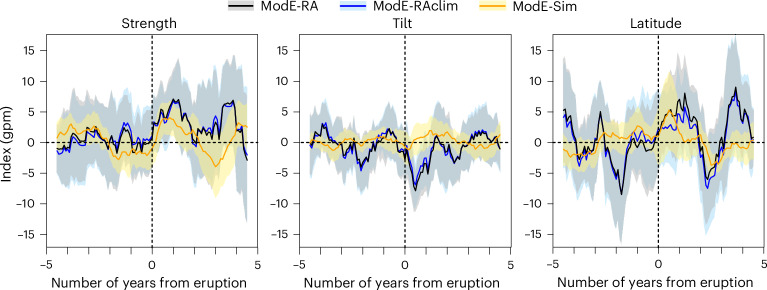
Volcanic effects on jet stream indices. Composite means of jet strength, tilt and latitude for 12 volcanic eruptions after 1680. The composited series are monthly anomalies (smoothed with a 12-month moving average) from the five years before eruption (where zero indicates three months after the eruption, when the first effects may become visible). The shading indicates the 95% confidence interval around the mean (solid line).

A second possible mechanism for forced variability in the jet includes volcanic eruptions. We selected all tropical or northern extratropical eruptions from Sigl et al.^45^ with at least −3 W m^−2^ of global radiative forcing and found 12 eruptions after 1680 (see Methods). We then composited the monthly index series for these eruptions, each expressed as anomalies from the previous five years. The results (Fig. 4) confirm a jet strengthening^46,47^, which is statistically significant in all three ModE-RA products (arising from the model alone and from the observations alone). A slight poleward shift is found, in agreement with previous research^47^, but is not statistically significant. We find a decrease in tilt, corresponding to a more zonal circulation. This response is statistically significant in ModE-RA and ModE-RAclim but not when modelled in ModE-Sim.

## Recent jet changes put into perspective

**Fig. 5:**
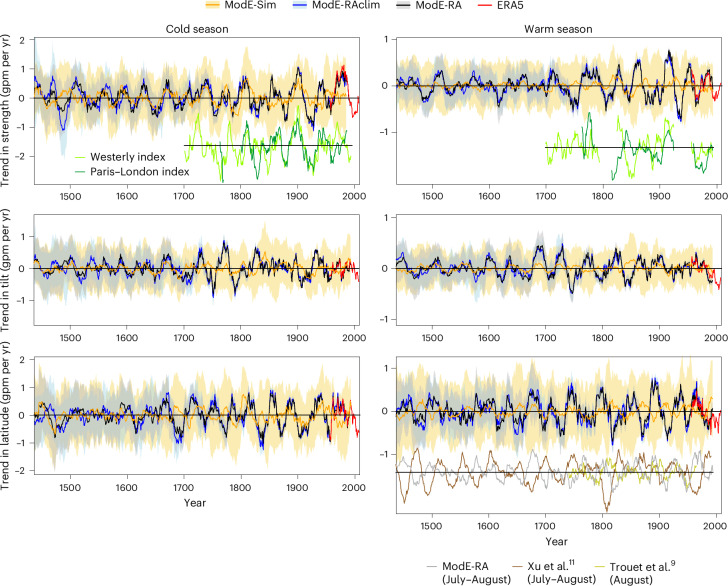
Trends in the jet stream. Thirty-year moving trends in the jet strength, tilt and latitude for the cold (left) and warm (right) seasons. Shading indicates ±2 standard deviations of the ensemble around the ensemble mean. Existing reconstructions that correspond to the jet strength^10^^,34^ (light and dark green lines) and to the jet latitude in the warm season^9,11^ (grey, brown and yellow-green lines) are also shown. These series were standardized before trend calculation and are shown with a line denoting zero but no scale.

**Extended Data Fig. 10:**
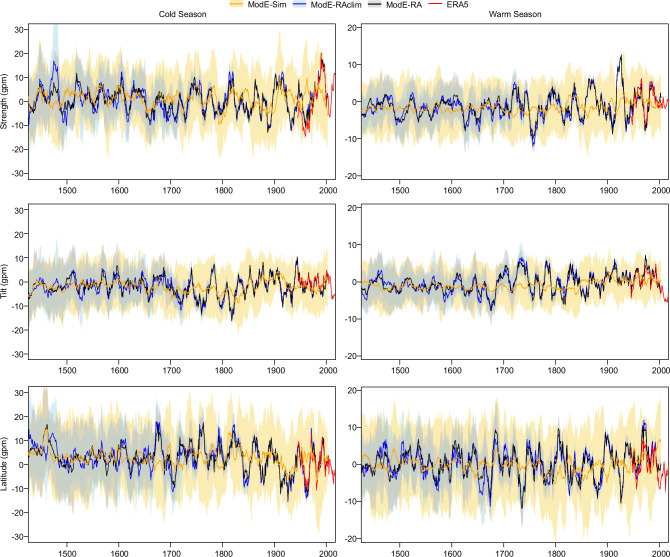
Decadal changes in jet stream indices. 10-year moving averages of jet strength, tilt, and latitude in the cold (left) and warm season (right). Shadings indicate ±2 standard deviations of the ensemble around the ensemble mean.

We analysed ten-year moving averages (Extended Data Fig. 10) and 30-year moving trends (Fig. 5) in the three jet indices. The results show that some recent changes, such as in the jet strength in the cold season during the 1960s to 1990s, are pronounced, but phases with strong trends occurred around 1900 and perhaps even in the fifteenth or sixteenth century^48^. The most recent trends in all indices are close to zero, although trends in the jet are expected in a future climate^4–7^. Trends in the jet strength during the cold season are confirmed by trends in the westerly index^10^ and Paris–London pressure gradient^34^. During the warm season, there is a qualitative agreement between our jet latitude index and the reconstruction by Trouet et al.^9^ but not with Xu et al.^11^ With respect to the ten-year averages, in the most recent 60 years both positive and negative deviations are at times strong, although they are inside the 95% confidence range of ModE-RA over the past 600 years. Given the importance of the jet stream for hydroclimate extremes, the absence of large changes in the jet indices indicates that thermodynamic, not dynamic, causes must be mostly responsible for increasing trends in flood and drought frequency^35^.

## Methods

The atmospheric circulation is studied in monthly 500 hPa GPH fields from the ModE-RA family of reanalyses, which provide monthly global fields back to 1421. All analyses are performed separately on all members, unless otherwise noted. We focus on the Atlantic and Western European sector (30° W to 40° E, 35–75° N), which is relevant for the European climate. We deseasonalized the fields by subtracting the long-term mean annual cycle taken from the 1851–1950 period. To form indices for strength, tilt and position we used predefined orthogonal base patterns, which are sine curves as a function of the longitude or latitude, that is, varying between −1 and 1 (Fig. 1). The latitude-weighted regression coefficient is then used as the index. The approach is intuitive and does not depend on either the number of patterns or trends in the entire field. The interpretation is straightforward, which can be seen from the correlation with the 500 hPa zonal wind data in Fig. 1 and Extended Data Fig. 2. Furthermore, the same patterns can be used over time and across datasets. Principal component analysis gave very similar results (Extended Data Fig. 3). It was performed on the monthly anomalies of one member of ModE-RA, 1851–1950. The first three PCs can be compared to strength, tilt and latitude. The time series obtained by projecting the deseasonalized fields of all products (including ERA5) onto these patterns correlate well with the strength, tilt and latitude obtained with the predefined patterns. However, in contrast to our predefined patterns, the PC patterns depend on the time period and dataset used, and interpretation can be rather ambiguous.

For reconstructing the blocking frequency from 1728 onwards, we calculated the seasonal frequencies of the CAP9 weather types. These were used as predictors in a multiple regression model that was calibrated with the seasonal blocking frequency from ERA5, 1940–2023. We also reconstructed time series for blocking averages in the regions 48–58° N/10° W to 20° E and 52–62° N/0–26° E for the cold and warm seasons, respectively. For this we used a backward selection approach to remove weather types that were not significant at *P* = 0.05. Reconstructions were evaluated using a leave-one-out approach within ERA5 and against 20CRv3. The time series also show a good agreement with 20CRv3 for decadal variability (Extended Data Fig. 6). The FPI is explained in detail in ref. ^35^, which also provides the code used to calculate the index. For the analysis of precipitation and temperature in 1801, we defined the Swiss part of the Rhine catchment in Basel as all pixels (at 1 × 1 km resolution)^41^ north of 46°33′ N, that is, the latitude of the Gotthard Pass. The area encompasses 31,648 km^2^.

For June–August 1741 we reconstructed daily SLP fields using an analogue approach exactly as described in ref. ^49^. It is based on daily pressure series from Leiden (1740–1750), London (1731–1750), Montpellier (1738–1748), Berlin (1738–1743), Nuremberg (1732–1743), Uppsala (1731–1750) and Padua (1731–1750) that were deseasonalized and standardized. The seven locations were also extracted from the ERA5 daily mean SLP, deseasonalized and standardized. Then, for each day in June–August 1741, the closest analogue day in the ERA5 period was found on the basis of the Euclidean distance. For the daily SLP the approach was tested by reconstructing June–August 1940 data while withholding this year from the pool of analogues. From the analogue days we also reconstructed the daily blocking frequency and averaged it over the June–August period.

We compare our jet indices with other products both spatially and as times series. For the latter, the SPEI reconstructions by Freund et al.^30^ were averaged over the region 10° W to 32° E, 46–64° N. The scPDSI reconstructions by Büntgen et al.^37^ refer to the region 45–53° N, 6–20° E.

Volcanic eruption composites were generated by selecting all tropical or northern extratropical eruptions from Sigl et al.^45^ with at least −3 W m^−2^ global radiative forcing. They were assigned the following eruptions months (using January for unknown eruptions, as denoted with an asterisk): January 1695*, June 1783, January 1809*, April 1815, January 1831*, January 1835, December 1861, August 1884, October 1912, March 1963, April 1982 and June 1991. We then considered the time periods from five years before to five years after the eruption and subtracted for each eruption the average of the five years before the eruption. The resulting monthly series were then filtered with a 12-month moving average. The confidence interval for the composite mean was calculated as two standard errors divided by the square root of the number of eruptions.

## Online content

Any methods, additional references, Nature Portfolio reporting summaries, source data, extended data, supplementary information, acknowledgements, peer review information; details of author contributions and competing interests; and statements of data and code availability are available at 10.1038/s41561-025-01654-y.

## Data Availability

The ModE-RA, ModE-RAclim and ModE-Sim data (Valler et al.^15^) can be downloaded from DKRZ at https://www.wdc-climate.de/ui/entry?acronym=ModE-RA. ERA5 reanalysis data are available from the Copernicus Climate Change Service Data Store. The Old World Drought Atlas is available from the NOAA Paleoclimatology website at https://www.ncei.noaa.gov/access/paleo-search/study/19419 (downloaded 9 November 2023, last accessed 5 December 2023), the PHYDA dataset can be downloaded from https://zenodo.org/records/1198817 (downloaded 9 November 2023, last accessed 5 December 2023). The data for the SPEI reconstruction by Freund et al.^30^ are available via figshare at 10.26188/21988628.v1 (downloaded 9 November 2023, last accessed 5 December 2023). The precipitation reconstruction by Pauling et al.^29^ can be retrieved from the NOAA Paleoclimatology website at https://www.ncei.noaa.gov/pub/data/paleo/historical/europe/pauling2006precip/ (last accessed 5 December 2023). The SPI reconstruction by Glaser and Kahle^36^ is available via Zenodo at https://zenodo.org/records/3405167 (downloaded 9 November 2023, last accessed 5 December 2023). The flood series on the Rhône River at Beaucaire (1816–2016) is available from https://www.plan-Rhone.fr/publications-131/actualisation-de-lhydrologie-des-crues-du-Rhone-1865.html?cHash=5628938abe287dc9ca390dad7373ae0e (data downloaded: 26 April 2020, last access: 5 December 2023). The CAP9 weather types are available via the BORIS Portal at 10.48350/195666 (last accessed 23 October 2024)^50^. The blocking reconstructions are also available from the BORIS Portal at 10.48620/36384 (last accessed 11 December 2024)^51^. The daily precipitation and temperature reconstructions for Switzerland^41^ are available via PANGAEA at 10.1594/PANGAEA.950236 (last accessed 23 October 2024)^52^. The daily weather diary from Haller, spanning 1545–1576, can be downloaded from Pfister and co-workers^42^. The jet reconstructions by Trouet et al.^9^ and Xu et al.^11^ are available from the NOAA Paleoclimatology website at https://www.ncei.noaa.gov/products/paleoclimatology.

## Code availability

The code to used to calculate the jet indices, as well as the input files and the calculated jet indices from ModE-RA, ModE-Sim and ModE-RAclim, is available via figshare at 10.6084/m9.figshare.28001564.v1 (last accessed December 2024)^53^. Extended Data Figure 7 was generated using ClimeApp (https://mode-ra.unibe.ch/climeapp/)^54^.
